# Lactate dynamics at the *Pseudomonas aeruginosa*–host interface: bacterial metabolism, host defense, and emerging links to histone lactylation

**DOI:** 10.3389/fimmu.2026.1979311

**Published:** 2026-10-12

**Authors:** Yi Jian, Zhijie Wu, Yuqiang Liang, Jin Cao, Mei Li, Fashuang Yang, Ke Wang

**Affiliations:** 1 Department of Pulmonary and Critical Care Medicine, The First Affiliated Hospital of Guangxi Medical University, Nanning, Guangxi, China; 2 Department of Geriatrics, Jinsha County People’s Hospital, Bijie, Guizhou, China

**Keywords:** bacterial metabolism, biofilm, H3K18la, histone lactylation, host defense, lactate dynamics, *Pseudomonas aeruginosa*

## Abstract

At the *Pseudomonas aeruginosa*–host interface, lactate is both a host immunometabolic product and a preferred bacterial carbon source. Its local concentration reflects host production, bacterial utilization, transport, and clearance rather than inflammation alone. *P. aeruginosa* uses D- and L-lactate through stereospecific, conditionally regulated systems. It preferentially consumes lactate in human burn-wound exudate, and this capacity contributes to early intracellular survival in macrophages. Lactate-associated acidity also increases bacterial vesicle release and quinolone signaling, weakening antimicrobial-peptide activity independently of chromatin modification. On the host side, bacterial L-lactate utilization and virulence-factor-associated suppression of epithelial glycolysis lower epithelial lactate and histone H3 lysine 18 lactylation (H3K18la). These changes are linked to disrupted RNA regulation and impaired ciliary clearance. In macrophages, the quorum-sensing signal 2-aminoacetophenone (2-AA) reprograms bioenergetics, autophagy, and lipid metabolism, promoting tolerance and intracellular persistence. A separate hypothesis connecting 2-AA to increased macrophage lactate and H3K18la is supported only by a supplement abstract and requires full-length validation. Histone lactylation is therefore an emerging extension of this biology. This narrative review follows two linked strands: bacterial lactate utilization and lactate-associated changes in host defense. It distinguishes established metabolic effects from proposed lactylation-dependent mechanisms and considers potential therapeutic directions and their trade-offs. These include targeting bacterial lactate utilization, airway acidity, host transport or modification enzymes, and 2-AA-associated tolerance. Any benefit must be assessed against the possibility of supplying bacterial nutrients or worsening host injury.

## 1 Introduction

*Pseudomonas aeruginosa* is a major cause of hospital-acquired pneumonia, chronic airway infection, and wound- and device-associated infection. Its persistence depends on metabolic plasticity, respiratory flexibility, biofilm growth, and adaptation to oxygen, nutrients, and host-derived stress, alongside classical virulence (1–4). Longitudinal evidence from cystic fibrosis shows lineage-specific adaptation rather than a single chronic-infection state (5–7). These processes alter carbon availability for both host cells and bacteria, placing lactate at the center of their interaction.

Infected airways contain millimolar lactate, and concentrations rise with inflammation (8–11). *P. aeruginosa* also takes up and oxidizes both lactate enantiomers through conditionally regulated systems. In human burn-wound exudate, it consumes lactate in preference to glucose (12–14). Elevated lactate and active bacterial consumption can therefore coexist. Inflammatory-cell glycolysis may replenish the extracellular pool faster than bacteria remove it, so bulk concentration reflects their net balance. The distinction has functional consequences. Lactate-associated acidity can weaken antimicrobial-peptide activity (Section 4.1), while bacterial utilization supports growth and intracellular survival.

Lactate supplies nutrients, reflects host glycolysis, and contributes to host signaling and chromatin regulation. Neither its abundance nor its consumption alone predicts the direction of host defense (15, 16). Two bacterial activities dominate the current literature. First, utilization through the L-lactate dehydrogenases LldD and LldA lowered epithelial lactate and histone H3 lysine 18 lactylation (H3K18la). A full-length airway study linked these changes to impaired mucociliary defense (17). Second, 2-aminoacetophenone (2-AA), a signal controlled by the multiple virulence factor regulator (MvfR), reprograms macrophage bioenergetics, autophagy, and lipid metabolism (18, 19). A supplement abstract proposes an additional lactate/H3K18la hypothesis in macrophages (20). These findings do not establish reciprocal branches of one validated pathway.

A change in lactate does not establish protein lactylation. We use “lactate-associated” when a study measures or alters lactate without showing that lactylation is the necessary mediator. Mechanistic interpretation requires a defined bacterial activity, host-cell lactate, a specified modification readout, and a defense endpoint (17). Here, host defense encompasses airway mucociliary clearance, macrophage antibacterial and tolerance programs, and autophagy. Bacterial burden, clearance, and persistence are infection outcomes. Tolerance limits infection-induced tissue damage and is distinct from microbial killing.

This review asks how lactate availability at the *P. aeruginosa*–host interface shapes bacterial adaptation and host defense, and where histone lactylation fits. Section 2 establishes the shared source–sink and compartmental framework. The bacterial strand follows regulated lactate utilization in Section 3.1 and its fitness consequences in Section 4.1. The host strand follows epithelial depletion and macrophage reprogramming in Sections 3.2–3.3, then their defense outcomes in Sections 4.2–4.3. Sections 5 and 6 examine mechanistic boundaries and potential therapeutic directions.

## 2 Lactate availability at the *Pseudomonas aeruginosa*–host interface

This traditional narrative review synthesizes literature on *P. aeruginosa* lactate metabolism, host lactate remodeling, and lactylation-associated defense. The original targeted searches covered PubMed, Web of Science, and Scopus through 23 August 2026 and included reference tracking. Greatest weight was given to studies linking a defined bacterial activity to a host-cell or functional outcome. The two editor-suggested 2026 articles were assessed separately during this revision. **Table 1** defines evidence tiers T1–T3, which distinguish paired infection evidence, studies of one side of the interface, and contextual or abstract-level reports.

**Table 1:** Representative studies linking *P. aeruginosa*-associated lactate dynamics to host responses.

Study/setting	Bacterial activity or interface feature	Lactate direction	Host lactylation or response	Host-defense consequence/infection outcome	Representative observation	Evidence tier	Interpretive boundary
Airway epithelium/mouse (17)	LldD/LldA-dependent L-lactate utilization + suppression of host glycolysis	Host ↓	H3K18la ↓; YTHDF1–DNAH5 pathway ↓	Ciliary beating and mucociliary clearance ↓; bacterial burden ↑	Bacterial mutant, lactate rescue, downstream perturbation, and *in vivo*/*in vitro* endpoints	T1	Single airway model; p300 intervention is not lactylation-specific; H3K18la assessed mainly by antibody-based assays and not extrapolable to the lactylome
2-AA-tolerized macrophages (18, 19, 80–82)	Separate bioenergetic and autophagic/lipid-metabolic programs	Not measured	HDAC1/H3K18ac, autophagy, lipid metabolism, ATP/acetyl-CoA; lactylation not measured	Tolerance, impaired clearance, and persistence	Full-length studies establish upstream and parallel host-remodeling pathways	T2	Do not establish lactate or H3K18la involvement; reduced acetyl-CoA or H3K18ac does not predict H3K18la, because the two modifications depend on distinct donor pools
Supplement abstract: proposed 2-AA branch (20)	Proposed glycolytic shift, LDHA-associated lactate, and GTPSCS–CBP/p300 involvement	Proposed host increase	Proposed H3K18la increase	Proposed association with tolerance and intracellular persistence	Research hypothesis based on a 2026 supplement abstract	T3	Full-length methods, sample sizes, and effect sizes unavailable; lactylation-specific necessity unestablished; requires full-length reporting and independent validation
Bronchiectasis airway; acidic-environment axis (66, 67)	Lactate-adjusted acidity → OMV release → HHQ hyperproduction; OMV-driven IRF3–IFN-β activation	Airway ↑	Not measured (non-lactylation mechanism)	LL-37 bactericidal activity ↓; antimicrobial-peptide resistance; intracellular survival and lung burden ↑	Airway lactate and LL-37 elevated in bronchiectasis, most in *P. aeruginosa* infection; OMVs increased under acidic conditions; EBC pH falls with disease severity	T2	Patient sampling plus *in vitro* mechanism; effect on host chromatin not assessed
Pathogen-side metabolism (13, 14, 55, 56)	LdhA; *lldPDE* (LldP/LldD/LldE); separately encoded *lldA*; LldR/LldS	Bacterial production, uptake, or oxidation	Not measured	D- and L-lactate utilization; D-lactate cross-feeding; early intracellular recovery	Isomer- and condition-dependent functions; LldD/LldA contribute in a short-term macrophage assay	T2	No host lactate or Kla measurement; cross-feeding not shown in patient tissue
Human burn-wound exudate (12)	Lactate used as a preferred substrate ex vivo	Matrix ↓	Not measured	Bacterial growth and transcriptional response	>96% lactate consumed after 24 h; glucose fell ~10.3%	T2	Ex vivo human matrix; no cellular localization or host Kla
CF sputum cohorts (8, 11, 28)	Mixed inflammatory host pool; bacterial uptake not traced	Variable	Not measured	Infection-ecology context	Millimolar lactate; association with neutrophil abundance; divergent treatment and glucose-load responses	T2	Observational bulk sample; host and pathogen sources and intra-/extracellular pools are mixed; the two treatment cohorts differ in design and should not be merged into one trajectory
Contextual: *S. aureus* biofilm (114)	Bacterial lactate inhibits HDAC11	↑	HDAC11 inhibition, unchecked HDAC6 activity, H3 acetylation, IL-10; not Kla	Persistent-infection immune reprogramming	Lactate–epigenetic host response	T3	Different pathogen and a non-lactylation mechanism
Contextual: contrasting sepsis states (122, 123)	Inflammatory versus immunosuppressive states	↑ or ↓	H3K18la direction varies with cell/state	Secretory autophagy/endothelial injury versus antibacterial autophagy	Two peer-reviewed studies illustrate cell- and stage-dependence	T3	Not *Pseudomonas*-specific and not demonstrated within one tissue/model

T1 denotes a full-length study measuring a bacterial pathway, host lactate, and a defense endpoint in *P. aeruginosa* infection. T2 denotes full-length evidence from only the bacterial or host side. T3 denotes evidence from other pathogens or systems, contextual clinical evidence, or abstract-level reports. These tiers describe relevance and completeness of the mechanistic evidence, not clinical efficacy. Arrows indicate reported changes within the specified setting; changes in the abstract-level row are proposed and require validation. CF, cystic fibrosis; EBC, exhaled-breath condensate; IL-10, interleukin-10. Other protein and modification symbols follow the definitions in the main text.

Lactate availability reflects host production, bacterial production and utilization, host-cell uptake, transport between compartments, and tissue clearance. A sample measures the pool remaining after these processes have acted. Interpretation therefore requires the source, the sink, and the sampled compartment to be specified.

### 2.1 Host sources and the bacterial sink

Host lactate at an infection site comes chiefly from glycolytic immune cells. Neutrophils rely heavily on glycolysis and are a major source in purulent inflammation. Their lactate can also promote mobilization through endothelial G protein-coupled receptor 81 (GPR81, also called hydroxycarboxylic acid receptor 1, HCAR1) (9). Macrophage glycolysis and hypoxia-inducible factor 1α signaling vary with stimulus and stage. Lactate output consequently depends on the initiating signal, nutrient environment, and stimulation duration, rather than lineage alone (21–26). In airway epithelial cells, *P. aeruginosa* infection suppressed glycolysis and lowered intracellular lactate (17). Host cells within the same lesion can therefore show different metabolic responses.

Changes in cellular composition further complicate bulk lactate measurements. Neutrophil influx, epithelial injury, macrophage recruitment, hypoxia, and bacterial adaptation alter the number and activity of lactate-producing cells. Bulk lactate can rise simply because a glycolytic population expands (2, 8–10, 21, 22, 27). Chronic airway samples combine neutrophil-rich secretions, injured epithelium, recruited macrophages, and adapted bacteria in proportions that change over time.

*P. aeruginosa* acts as a bacterial sink against these host sources. It uses both D- and L-lactate through conditionally regulated systems (13, 14) and preferentially consumes lactate in human-derived matrices (12) (Section 4.1). Bulk clinical measurements capture production and consumption together. In one cystic fibrosis exacerbation cohort, mean sputum lactate was approximately 3.0 mmol/L, with a standard deviation of 3.1 mmol/L (8). The dispersion exceeded the mean, and lactate fell after intravenous antibiotics. In a second cohort, L-lactate remained stable during elective treatment and correlated with neutrophil counts (11). An oral glucose load also raised sputum lactate (28). These differing trajectories should not be combined into a single treatment response. Antibiotics can remove a bacterial sink while reducing neutrophilic inflammation; perfusion and drainage also influence the balance. The glucose-load response further shows that host substrate supply affects the extracellular pool.

Neither low nor high lactate identifies bacterial consumption on its own. Flux attribution requires substrate-consumption measurements, pathway perturbation, or temporally resolved data, ideally paired with evidence of bacterial pathway expression. Sputum nutrient cues alter *P. aeruginosa* gene requirements, and standard laboratory media reproduce this physiology incompletely (13, 14, 29–32). The key distinction is between the measured net pool and the processes producing it.

### 2.2 Transport, clearance, and compartmentalization

Host transport separates extracellular exposure from intracellular lactate availability. Monocarboxylate transporters (MCTs) 1–4 carry lactate together with a proton. Net transport depends on lactate and proton gradients, transporter expression, and cellular metabolic state (33–35). Perfusion, exudate volume, and drainage also influence clearance (36, 37). Apical secretion can enlarge the airway-surface pool while intracellular lactate falls. Conversely, uptake can support metabolism without an increase in production.

Compartment-specific measurements illustrate this distinction. In polarized cystic fibrosis bronchial epithelial cultures, 6 h of PAO1 exposure raised airway-surface lactate from 3.3 to 14.9 mmol/L. The medium contained 5 mmol/L glucose; MCT1/2 inhibition attenuated the rise, and MCT2 localized predominantly to the apical membrane (38). This extracellular increase differs from the intracellular decline in the airway H3K18la study (17). The studies examined different compartments, cellular backgrounds, strains, and time points, so their findings are not directly opposing measurements. Isotope tracing also shows that extracellular lactate can provide substantial oxidative carbon (39–41). Uptake and production are therefore distinct readouts.

Lactate accumulation and acidification require separate evaluation because pH also reflects proton clearance, CO_2_, and buffering capacity (42, 43). Local immune and bacterial processes further shape the infection environment. In *P. aeruginosa* pleural infection models, bacterial cyclic di-guanosine monophosphate signaling regulated neutrophil extracellular trap formation, and deoxyribonuclease inhibited early biofilm development (44, 45). These findings provide infection-environment context rather than direct measurements of lactate transport.

The airway lumen, biofilm layers, host-cell cytosol, and nucleus are distinct compartments. Oxygen, nutrient, and iron gradients determine where bacteria oxidize lactate and where host cells produce it (14, 46–48). Activated polymorphonuclear leukocytes consume oxygen and help create anoxic niches containing slowly growing bacterial aggregates (49–54). Sputum reports a predominantly extracellular mixed-cell pool, whereas tissue homogenate combines extracellular and intracellular material. Neither directly reports cytosolic or nuclear lactate availability in an individual host cell. **Figure 1** summarizes these source–sink, transport, and compartmental boundaries.

**Figure 1:**
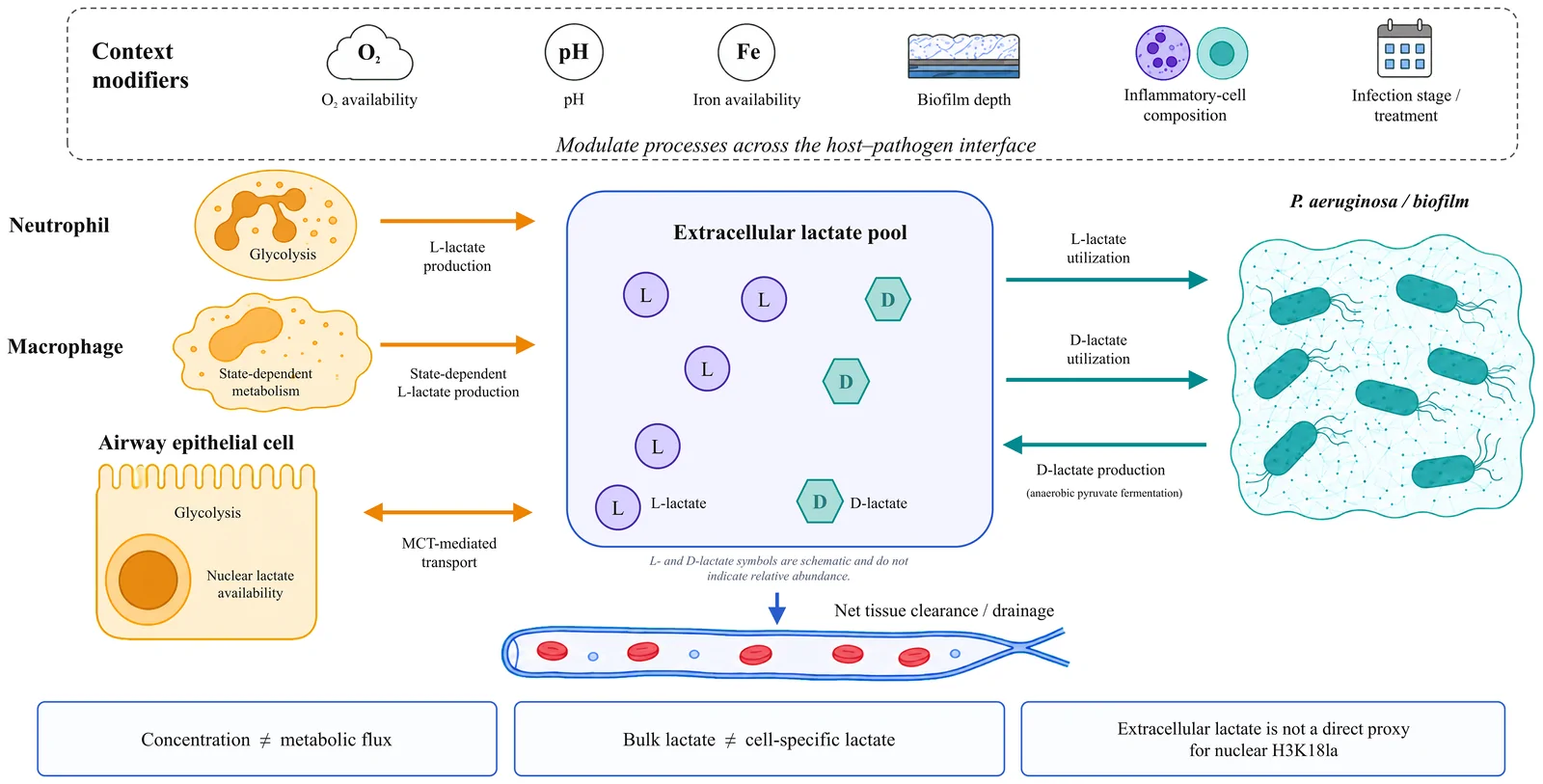
Source–sink ecology and compartmental boundaries of lactate at the *Pseudomonas aeruginosa*–host interface. Host neutrophils, macrophages, and airway epithelial cells contribute L-lactate to infection-site pools. *P. aeruginosa* utilizes both L- and D-lactate and generates D-lactate during anaerobic pyruvate fermentation. Monocarboxylate transporter (MCT)-mediated transport separates extracellular from intracellular availability; tissue clearance and drainage further shape the extracellular pool. Oxygen, pH, iron, biofilm depth, inflammatory-cell composition, and infection stage or treatment modify these processes. L- and D-lactate symbols are schematic and do not indicate relative abundance. Bulk concentration does not directly report nuclear availability. **Table 2** specifies the measurement boundaries.

## 3 How *Pseudomonas aeruginosa* consumes and reshapes host lactate

*P. aeruginosa* changes host lactate availability through substrate utilization and host-cell reprogramming. Section 3.1 addresses bacterial uptake and oxidation. Sections 3.2 and 3.3 examine epithelial depletion and macrophage metabolic changes, respectively. These activities can coexist, but their effects on host lactate and defense require separate evaluation.

### 3.1 Stereospecific utilization and its conditional regulation

Bacterial lactate utilization depends on stereospecific enzymes and respiratory conditions. The repertoire includes the predicted permease LldP and the enzymes LdhA, LldD, LldE, and LldA. During anaerobic fermentation, LdhA reduces pyruvate to D-lactate; LldE supports D-lactate oxidation. LldD and LldA independently oxidize L-lactate under aerobic conditions and transfer electrons into the quinone pool. The *lldP*, *lldD*, and *lldE* genes form the *lldPDE* operon, whereas *lldA* occupies a separate locus (13, 14, 55). This organization couples substrate stereochemistry to respiratory conditions.

LldD and LldA provide overlapping capacity under different conditions. Reporter assays showed *lldPDE* responses within the lower tested L-lactate range, while *lldA* responded at higher concentrations and differed across macrocolony depth (14). LldR represses *lldPDE*, and the L-lactate-responsive activator LldS is required for *lldA* expression. Low iron preferentially induces *lldA*, whereas glycolate mainly suppresses *lldPDE* (13, 14, 56). Chemical and spatial conditions therefore determine which system is deployed.

These observations derive from defined media and laboratory biofilms. The contribution of each enzyme within infected human tissue remains unresolved. The reporter data nevertheless establish conditional, rather than constitutive, lactate utilization. Environmental lactate measurements are therefore most informative when paired with evidence of pathway expression. **Figure 2** summarizes the pathways and their regulation.

**Figure 2:**
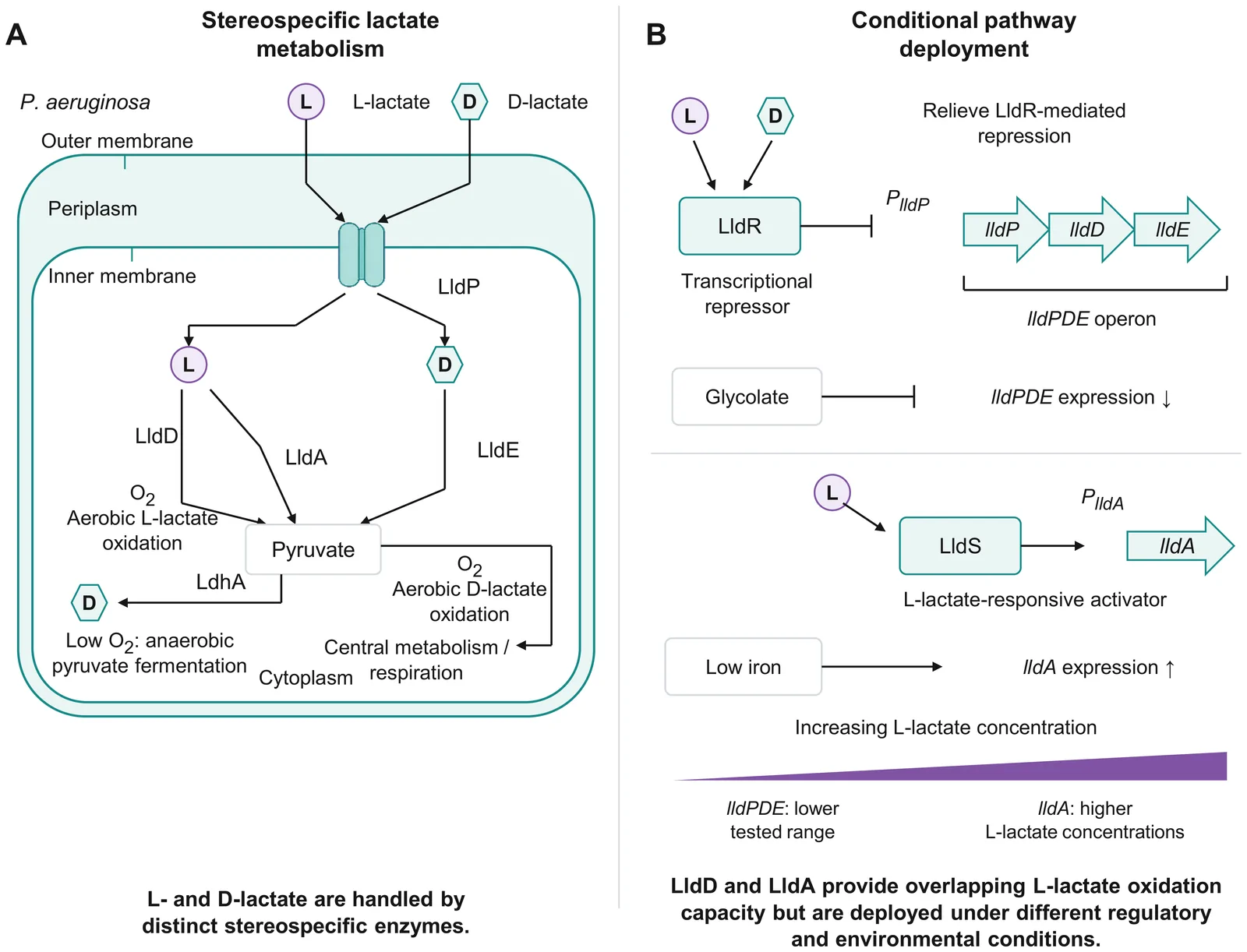
Stereospecific lactate metabolism and conditional pathway deployment in *Pseudomonas aeruginosa*. **(A)** LdhA produces D-lactate during anaerobic pyruvate fermentation, and LldE oxidizes D-lactate. LldD and LldA independently support aerobic L-lactate oxidation; LldP is the predicted inner-membrane lactate permease. Protein names are roman and gene names italic. The outer membrane, periplasm, inner membrane, and cytoplasm are labeled separately; the panel key defines the L- and D-lactate symbols. **(B)** The *lldPDE* and *lldA* systems are conditionally and differentially regulated. L- and D-lactate relieve LldR-mediated repression of *lldPDE*, while LldS activates *lldA* in response to L-lactate. Glycolate suppresses *lldPDE*, whereas low iron favors *lldA*. The two oxidation systems respond over different tested L-lactate ranges and biofilm depths. Reporter data derive from defined media and laboratory biofilms. The relative contribution of each enzyme in infected human tissue remains unresolved. These bacterial findings alone do not establish host lactate depletion or altered histone H3 lysine 18 lactylation (H3K18la).

### 3.2 Depletion of the host epithelial pool

On the host side, bacterial utilization can deplete the epithelial lactate pool. Wang et al. linked LldD/LldA-dependent utilization to virulence-factor-associated suppression of glycolysis in airway epithelial models (17). Increased bacterial consumption and reduced host production acted in the same direction. Wild-type infection lowered epithelial lactate and H3K18la relative to the Δ*lldD*Δ*lldA* mutant. The study connected these molecular changes to ciliary activity and bacterial burden, extending beyond bacterial growth measurements alone.

The data do not quantitatively separate bacterial uptake from suppression of host glycolysis, and the bacterial factors causing that suppression remain unidentified. The mutant probes utilization, whereas lactate supplementation tests metabolic reversibility (17). The carbon balance is therefore qualitative. Nevertheless, bacterial and host-directed perturbations converge on the epithelial phenotype, linking substrate availability to host defense. Section 4.2 examines the consequences for mucociliary clearance.

### 3.3 2-AA-driven reprogramming of host metabolism

Macrophage reprogramming changes host metabolism without directly consuming the host lactate pool. Wang et al. review how bacterial infection alters macrophage glycolysis and metabolite-dependent immune regulation across pathogens (57). This provides context for the *P. aeruginosa* studies, rather than validation of a 2-AA–H3K18la mechanism. Full-length studies identify two distinct 2-AA-associated programs (**Table 1**).

One program links 2-AA to histone deacetylase 1 (HDAC1)-dependent regulation and reduced histone H3 lysine 18 acetylation (H3K18ac) at the *Scd1* and *Beclin1* promoters. Changes in stearoyl-CoA desaturase 1 (SCD1), Beclin1, and unc-51-like kinase 1 (ULK1) accompanied altered autophagy and lipid metabolism, impairing intracellular clearance (18). The other program involves peroxisome proliferator-activated receptor γ coactivator 1α (PGC-1α), estrogen-related receptor α (ERRα), and mitochondrial pyruvate carrier 1 (MPC1). 2-AA weakened PGC-1α/ERRα interaction and MPC1 expression, reducing mitochondrial pyruvate import, ATP, and acetyl-CoA while altering cytokine responses and clearance (19). MvfR broadly controls quinolone signaling and persistence-associated virulence (58–60). Moreover, 2-AA feeds back on MvfR and perturbs mitochondrial function in host tissue (61, 62). Thus, MvfR-dependent phenotypes cannot all be assigned to 2-AA.

The two programs are parallel evidence streams rather than one linear pathway. Neither study measured lactate, lactate dehydrogenase A (LDHA), or H3K18la. The 2-AA state also changes over time. ATP and acetyl-CoA may rise after short exposure, then decline with mitochondrial respiration as tolerance develops (19). In the autophagy–lipid model, PA14 or exogenous 2-AA reduced microtubule-associated protein 1 light chain 3B puncta, ULK1, Beclin1, and SCD1. Palmitoyl-CoA or stearoyl-CoA improved intracellular clearance (18). Together, these findings connect established 2-AA responses to mitochondrial capacity and the substrates and machinery needed for intracellular bacterial handling.

A supplement abstract proposes a research hypothesis linking 2-AA to increased LDHA-associated lactate and H3K18la through GTP-specific succinyl-CoA synthetase (GTPSCS) and CREB-binding protein (CBP)/p300 (20). This proposed tolerance–persistence pathway requires full-length reporting and independent validation (**Table 1**).

## 4 What lactate does at the interface

The functional consequences of lactate changes differ between the bacterium and its host. Section 4.1 examines bacterial nutrition and the local chemical environment. Sections 4.2 and 4.3 examine epithelial clearance and macrophage defense, respectively. Each strand requires its own functional endpoint and evidence boundary.

### 4.1 Bacterial fitness and adaptation

Lactate first supports b*a*ct*eria*l fitness as a growth substrate. In ex vivo human burn-wound exudate, bacteria consumed more than 96% of available lactate within 24 h, while glucose fell by approximately 10.3% (12). Lactate was therefore preferred in this human-derived matrix. Utilization also contributes to survival inside host cells. Both L-lactate-oxidation reporters were expressed during RAW264.7 macrophage infection, and deleting *lldD*, *lldA*, or *lldS* reduced bacterial recovery at 3 h (14). The double mutant showed no additive effect, indicating that both enzymes contribute under the same short-term assay conditions. However, the compartment supplying lactate to intracellular bacteria remains undefined.

Lactate metabolism also organizes bacterial populations spatially. Laboratory biofilms support LdhA-dependent D-lactate production in low-oxygen regions and LldE-dependent oxidation in more oxygenated regions (13, 63–65). Production and consumption can therefore occur at different depths within one aggregate. Such cross-feeding has not been demonstrated in patient tissue. Antimicrobial exposure and biofilm maturation may also shift these boundaries. The laboratory findings nevertheless provide a physiological rationale for retaining both enantiomer-specific systems.

Lactate-associated acidity provides a further benefit to bacteria. Airway lactate and the cathelicidin LL-37 were elevated in bronchiectasis, most markedly in patients infected with *P. aeruginosa* (66). Experimentally imposed, lactate-adjusted acidic conditions increased bacterial outer-membrane vesicle (OMV) production. The vesicles induced 2-heptyl-4-quinolone (HHQ) hyperproduction. Positively charged HHQ interfered with LL-37 binding to the bacterial membrane, conferring peptide resistance (66). Exhaled-breath-condensate pH also fell with bronchiectasis severity. In experimental infection, lactic-acid-adjusted acidity increased OMV release and activated interferon regulatory factor 3 (IRF3)–interferon-β (IFN-β) signaling. These changes increased intracellular bacterial survival and lung bacterial burden (67). Here, the accumulated host-derived pool and associated acidity favor the pathogen without requiring histone lactylation.

Clinical observations also connect circulating quorum-sensing molecules with lactate, although they do not establish the airway mechanism. Mariano et al. studied 12 burn patients with septic shock and acute kidney injury receiving continuous kidney replacement therapy and CytoSorb hemoadsorption (68). Baseline lactate correlated with selected signaling molecules, including the *Pseudomonas* quinolone signal C7-PQS, with associations also reported in the *P. aeruginosa* bloodstream-infection subgroup. Some molecules decreased early during adsorption and partially rebounded later. This observational pilot study supports an association and extracorporeal removability, but establishes neither causal lactate regulation nor therapeutic efficacy.

Bacterial fitness can thus increase through lactate nutrition or through lactate-associated changes in the local environment, independently of chromatin modification. A rising bulk concentration does not imply absent bacterial utilization. Instead, active consumption and an extracellular pool permissive for persistence can coexist.

### 4.2 Epithelial mucociliary defense

The host epithelial strand links lactate availability to mucociliary defense. The airway study reported reduced lactate and H3K18la alongside lower YTH N^6^-methyladenosine RNA-binding protein 1 (YTHDF1) abundance (17). It also reported reduced N^6^-methyladenosine (m^6^A) methylation of dynein axonemal heavy chain 5 (DNAH5) mRNA and impaired ciliary function. Cell-based and animal experiments connected the bacterial metabolic perturbation to ciliary beating, mucociliary transport, inflammation, and bacterial burden. Mucociliary clearance requires coordinated beating, separation of mucus from the epithelial surface, and mucus detachment (69–71). It is therefore a direct component of airway defense.

YTHDF1 is an m^6^A reader that promotes translation of methylated transcripts, rather than a methyltransferase (72, 73). Reduced YTHDF1 implies altered recognition and translation of modified *DNAH5* mRNA; it does not itself establish reduced m^6^A deposition. Convergent m^6^A-sequencing, YTHDF1-binding, and polysome data identified DNAH5 as the downstream transcript (17). *DNAH5* encodes an axonemal dynein component required for motile cilia (74). The evidence therefore connects chromatin and RNA regulation at ciliary defense, without establishing direct control of m^6^A deposition by lactylation.

Distinct interventions support successive links in the epithelial model, with different specificity limits. The *ΔlldDΔlldA* mutant implicates bacterial L-lactate utilization. Lactate supplementation and respiratory-metabolism perturbation support metabolic reversibility, while C646-mediated p300 inhibition supports a modification-associated step. Conditional *Ythdf1* deletion increased bacterial burden and inflammation in mice, supporting the downstream reader function (17). These independent perturbations converge on successive biological levels, but no single intervention specifically tests every link. **Table 1** records the limitations, and **Figure 3** summarizes the model and its perturbations.

**Figure 3:**
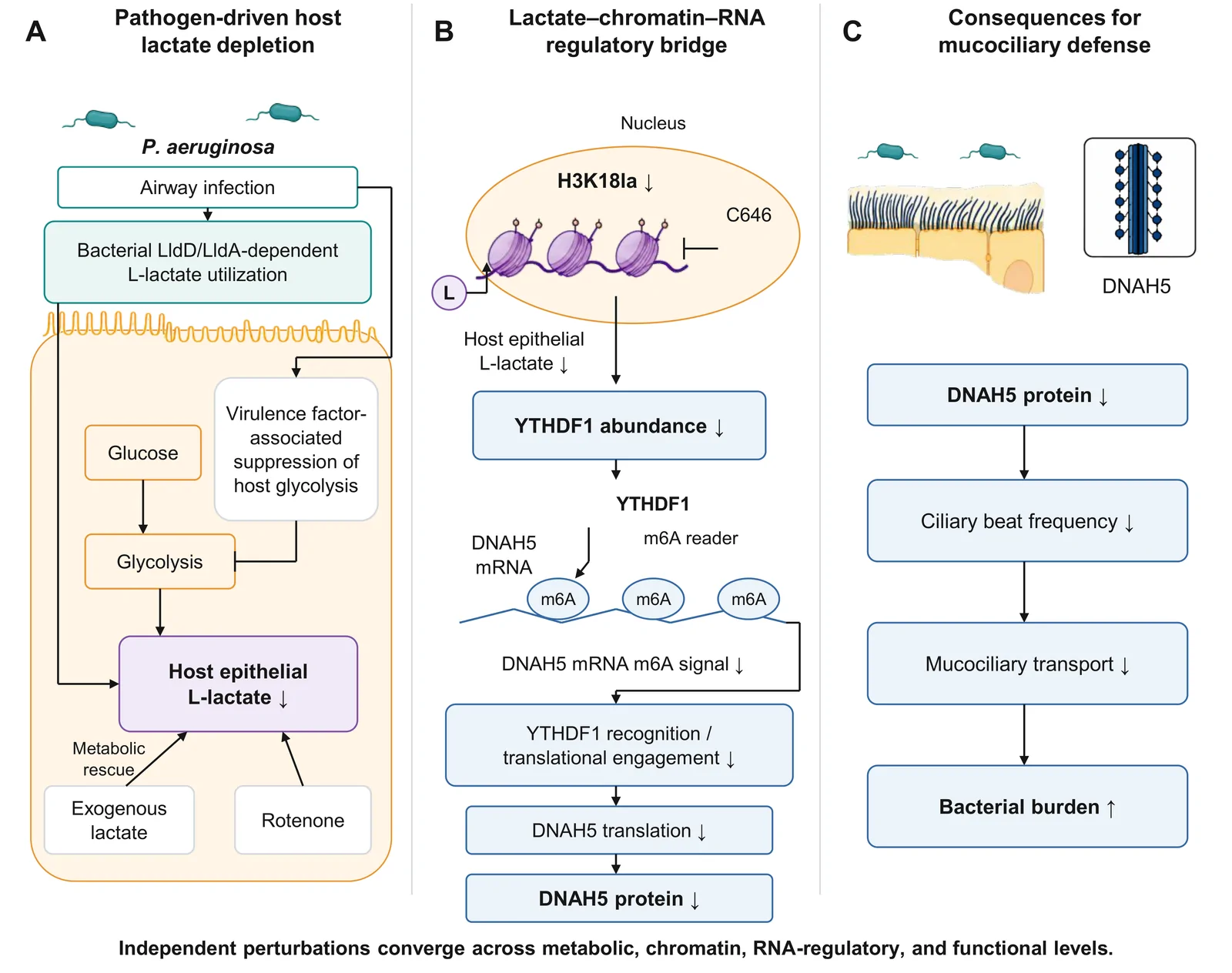
The airway epithelial lactate–H3K18la–YTHDF1–DNAH5 defense pathway. **(A)** Bacterial LldD/LldA-dependent L-lactate utilization increases the lactate sink while virulence factor-associated suppression of host glycolysis decreases the host source; together these lower epithelial L-lactate. **(B)** Reduced host lactate is associated with lower H3K18la and YTHDF1 abundance, alongside altered *DNAH5* mRNA m^6^A readout, reader engagement, and translation. **(C)** Reduced DNAH5 output is linked to impaired ciliary beating and mucociliary transport, with increased bacterial burden as the corresponding infection outcome. Exogenous lactate and rotenone provide metabolic-rescue evidence. C646-mediated p300 inhibition supports a modification-associated step but is not lactylation-specific. The *ΔlldDΔlldA* mutant probes bacterial utilization, while conditional *Ythdf1* deletion tests the downstream reader function (17). H3K18la denotes histone H3 lysine 18 lactylation. YTHDF1 is the YTH N^6^-methyladenosine RNA-binding protein 1 reader; DNAH5 is dynein axonemal heavy chain 5. m^6^A denotes N^6^-methyladenosine. YTHDF1 reads rather than installs this modification. The model therefore connects chromatin and RNA regulation without implying m^6^A deposition by lactylation. No single intervention establishes every molecular link.

Ciliary beat frequency and mucociliary transport directly measure epithelial defense. Bacterial burden also reflects replication and other tissue responses. Increased burden after conditional *Ythdf1* deletion therefore supports *in vivo* relevance without assigning every inflammatory change to the epithelial pathway (17). Impaired clearance could prolong pathogen–epithelial contact, but this feedback remains temporally unresolved. Other processes also affect epithelial defense. Airway-surface dehydration and acidification impair eradication and favor biofilm persistence (75–77). *P. aeruginosa* can damage cilia directly, and impaired ciliary function permits greater epithelial invasion (78). Thus, the lactate-associated pathway contributes to epithelial defense within a broader physiological setting; Section 6 considers the trade-offs of lactate rescue.

### 4.3 Macrophage tolerance and persistence

Full-length macrophage studies support 2-AA-associated metabolic remodeling and impaired clearance, without establishing a lactylation-dependent step. The autophagy–lipid study identified reduced SCD1-associated lipid biosynthesis and changes in Beclin1 and ULK1 (18). The bioenergetic study instead centered on PGC-1α/ERRα control of MPC1 and mitochondrial pyruvate entry (19). Both routes affect machinery that independently influences infection outcomes. For example, mitophagy involving prohibitin 2, presenilin-associated rhomboid-like protease, PGAM family member 5, and PTEN-induced kinase 1 increased resistance to *P. aeruginosa* (79). Earlier work also showed that 2-AA could limit inflammatory injury while permitting higher bacterial burden (80–82).

These findings distinguish resistance from tolerance. Resistance lowers pathogen load, whereas tolerance limits tissue injury at a given load. A 2-AA-associated state can therefore reduce acute damage while weakening clearance. Reduced cytokine output alone does not identify the mechanism of persistence. Macrophage antimicrobial functions depend on ATP supply, autophagic competence, lipid metabolism, and the duration of tolerance. Higher bacterial burden reflects weakened clearance in a tolerized host rather than global macrophage failure. The balance of benefit to host and pathogen may consequently change with infection stage.

Several metabolic defects were partly reversible in the experimental models. ATP improved MPC1/ERRα expression, inflammatory responses, and intracellular clearance. Palmitoyl-CoA or stearoyl-CoA improved clearance in the autophagy–lipid model. Conversely, the mitochondrial pyruvate carrier inhibitor UK5099 increased burden and reduced tumor necrosis factor α during infection with the more readily cleared *ΔmvfR* mutant (18, 19). These interventions support functional roles for the corresponding metabolic nodes without establishing mediation by H3K18la.

The lactate/H3K18la research hypothesis remains unvalidated (20); lactylation-specific necessity has not been established. Its scope may depend on repeated 2-AA exposure. Lactylation can accompany repair, whereas lactate- and interleukin-6-dependent inflammatory adaptation remain separable (83, 84). **Figure 4** distinguishes established programs from this hypothesis.

**Figure 4:**
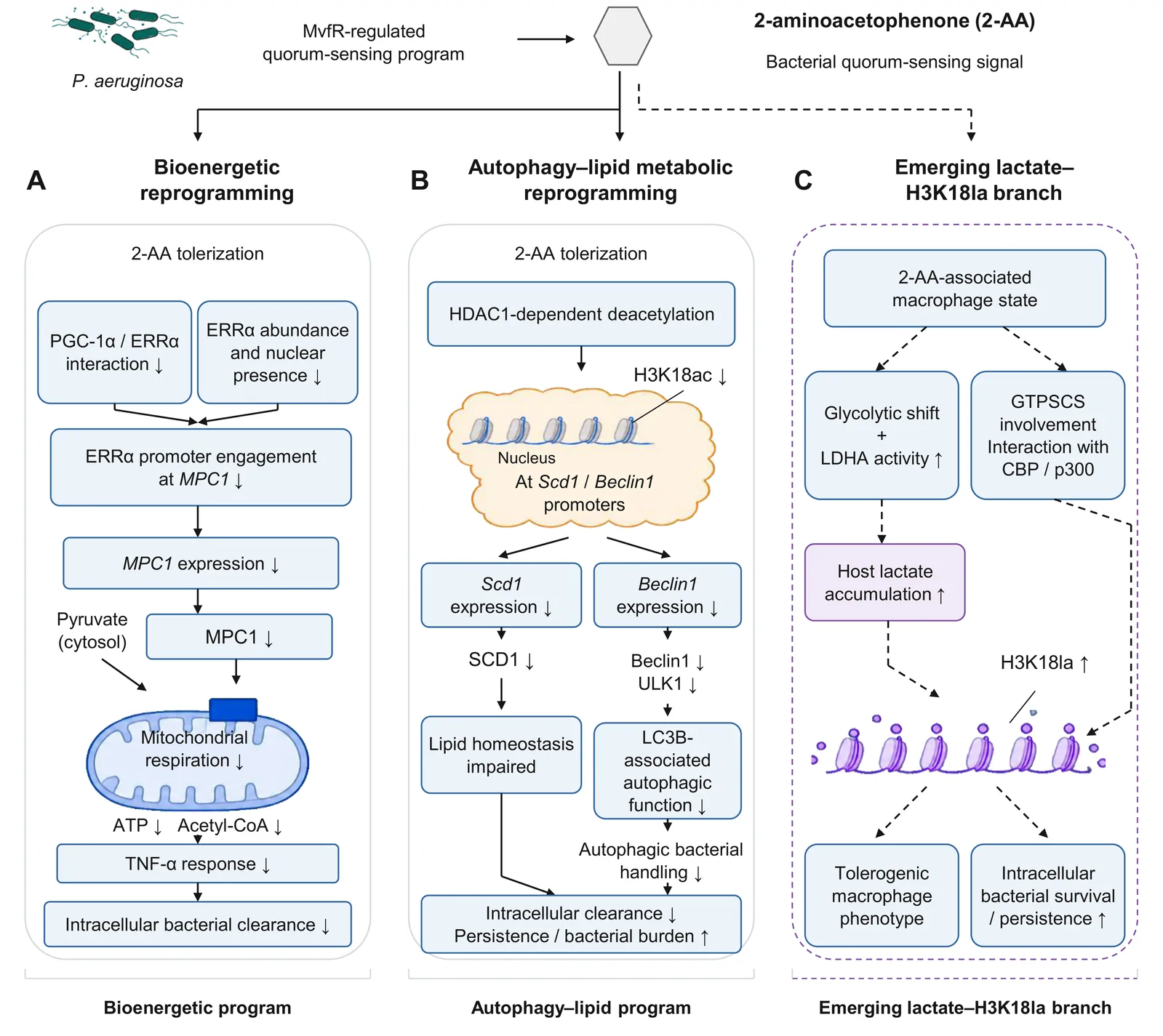
2-AA-associated macrophage reprogramming. The MvfR-regulated quorum-sensing signal 2-aminoacetophenone (2-AA) affects macrophage metabolism through distinct evidence streams. Panels **(A, B)** summarize full-length studies; panel **(C)** describes a research hypothesis. These panels should not be read as one validated linear pathway. **(A)** The bioenergetic program involves altered PGC-1α/ERRα regulation, lower MPC1 expression and mitochondrial pyruvate import, impaired respiration and ATP availability, and altered inflammatory responses. Intracellular bacterial clearance was reduced, and the phenotype depended on exposure stage (19). Mitochondrial pyruvate carrier inhibition and ATP supplementation affected this node in opposite directions. **(B)** The autophagy–lipid program involves HDAC1-dependent regulation, reduced histone H3 lysine 18 acetylation (H3K18ac) at the *Scd1* and *Beclin1* promoters, and impaired intracellular clearance (18). Changes in lipid metabolism and autophagy belong to this evidence stream. H3K18ac is an acetylation mark and does not establish lactylation. **(C)** Research hypothesis, not an established mechanism. A supplement abstract proposes increased LDHA-associated lactate and histone H3 lysine 18 lactylation (H3K18la), with GTPSCS–CBP/p300 involvement, in 2-AA-associated tolerance and persistence (20). Dashed borders and arrows denote the proposed status of these links. Full-length methods and independent validation are required before assigning a lactylation-dependent mechanism. MvfR, multiple virulence factor regulator; PGC-1α, peroxisome proliferator-activated receptor γ coactivator 1α; ERRα, estrogen-related receptor α; MPC1, mitochondrial pyruvate carrier 1; HDAC1, histone deacetylase 1; LDHA, lactate dehydrogenase A; GTPSCS, GTP-specific succinyl-CoA synthetase; CBP, CREB-binding protein.

## 5 Histone lactylation: an emerging extension

Direct histone-lactylation evidence in the *P. aeruginosa* studies discussed here centers on H3K18la. These studies do not establish site-specific, functionally validated non-histone lactylation in this infection. Chemical identity, donor and enzyme biology, and measurement specificity determine how far the evidence can be extended.

### 5.1 Chemical identity, donors, and enzymes

Lysine lactylation (Kla) encompasses chemically distinct L- and D-lactyl modifications. Functionally validated sites occur on histones and, in other systems, on DNA-repair, mitochondrial, and cytoskeletal-signaling proteins (85–90). L-lactyllysine (K_L_-la) is associated mainly with L-lactate-driven metabolism. D-lactyllysine (K_D_-la) can arise non-enzymatically from S-D-lactoylglutathione. N^ϵ^-carboxyethyllysine (Kce) is a distinct methylglyoxal-derived adduct. These species share the same nominal mass shift in conventional mass spectrometry and are not resolved by most antibodies (85, 91–96).

Isomer-resolved studies indicate that glycolysis-responsive histone lactylation in mammalian cells is predominantly K_L_-la. They also distinguish glyoxalase-derived, non-enzymatic D-lactylation from hypoxia-induced L-lactylation (97, 98). We use Kla for lysine lactylation, while treating a pan-Kla signal as an assay readout with unresolved site and isomer specificity. Such a signal cannot establish chemical identity in an infection sample. Likewise, bacterial D-lactate production or utilization does not identify host K_D_-la (13, 55).

Donor chemistry and enzyme identity impose a second constraint. Lactoyl-CoA is scarce in mammalian cells, and nuclear GTPSCS–p300 and acyl-CoA synthetase short-chain family member 2 (ACSS2)–lysine acetyltransferase 2A (KAT2A) provide reported donor-generation and transfer routes (99–102). Alanyl-tRNA synthetases AARS1 and AARS2 instead support an ATP-dependent route involving activated lactate, rather than requiring lactoyl-CoA (103, 104). Thus, lactate concentration alone does not define modification capacity across these different enzymatic routes.

Class I HDACs act as delactylases in some settings and catalyze lysine lactylation in others. Fixed writer–eraser labels are therefore misleading; enzyme perturbations require interpretation for the residue, chemical identity, and model studied (105, 106). Bacterial lysine lactylation is also enzymatically regulated (107). Bacterial lactylomes and host chromatin consequently represent related but distinct compartments in mixed samples.

### 5.2 Lactate dependence is not lactylation dependence

Lactate can alter host function without protein lactylation. Relevant routes include carbon oxidation, redox and pyruvate/lactate balance, MCT-mediated transport, HCAR1/GPR81 signaling, and pH (9, 33, 34, 108–113). The acidification–OMV–LL-37 pathway provides an infection-specific example acting on the pathogen rather than host chromatin (66). Timing further limits causal interpretation. Lactate responds rapidly to glycolysis and transport, whereas chromatin marks and transcriptional responses follow different kinetics. Parallel changes may therefore be causally linked or share an upstream driver, such as suppressed glycolysis.

Manipulating lactate and observing a functional change establishes a lactate-associated effect. Lactylation-specific interpretation additionally requires residue identification, perturbation of mark installation or removal, and linkage to a defense function. In the airway model, lactate rescue supports metabolic reversibility and p300 inhibition supports a modification-associated step; neither intervention is lactylation-specific (17). Similarly, the *Staphylococcus aureus* biofilm–HDAC11 mechanism supports lactate-dependent epigenetic regulation without establishing lactylation (114).

### 5.3 Matching measurement to claim

Measurement scale must match the claim. Total lactate does not distinguish host from bacterial sources, enantiomers, or intracellular from extracellular pools. Supernatant and airway-surface fluid report extracellular net balance; whole-cell lysates mix subcellular pools, and tissue homogenates additionally combine cell lineages and bacteria. Reduced intracellular epithelial lactate and increased apical secretion are therefore compatible (8, 11, 12, 17, 38). Histone-mark immunoassays require independent validation because off-target binding and neighboring marks can alter apparent site specificity (115, 116). Pan-Kla reactivity alone identifies neither the modified protein, site, nor isomer. Imaging, immunoprecipitation, site-specific mass spectrometry, and stable-isotope tracing improve spatial, association, site, and flux resolution, respectively, but each retains specific limitations (117–121). **Table 2** summarizes these interpretive boundaries.

**Table 2:** Interpretive scope and limitations of commonly used lactate and lactylation measurements.

Measurement	Directly supports	Does not resolve alone	Principal interpretive boundary
Total lactate (sputum, exudate, homogenate)	Overall concentration and temporal change	Host versus bacterial source; isomer; intra-/extracellular distribution	Bulk net pool; sputum is predominantly extracellular, whereas tissue homogenate mixes intra- and extracellular material. Opposing cell populations can cancel. Handling can distort inferred flux unless paired with tracing and rapid quenching (120, 121)
L-/D-lactate analysis	Enantiomer composition and change	Producing cell or subcellular localization	D-lactate cannot be equated with host K_D_-la
Pan-Kla immunoblot	Change in overall antibody reactivity	Modified protein, residue, isomer, or causal function	Does not establish chemical identity; conventional antibodies may not resolve isobaric adducts
H3K18la assay	Signal attributed to one histone site, subject to antibody validation	Other histone or non-histone lactylation	Cannot serve as a proxy for the entire lactylome; commercial histone-mark antibodies require independent validation because of off-target and neighboring-mark effects (115, 116)
Immunofluorescence/IHC	Cellular and spatial trend	Exact site or chemical identity	Requires lineage markers and a validated antibody
Immunoprecipitation	Association of a target with a Kla signal	Whether the target itself carries the modification	Association does not establish site assignment
Site-specific MS	Candidate modified protein and site; relative site quantification	Causal function	Confidence depends on peptide quality, isomer resolution, and sample purity (117, 118)
Stable-isotope tracing	Carbon fate and pathway contribution in an appropriate model	Species or cell source in a mixed host–pathogen sample	Mixed samples require source-resolved interpretation (119)

Kla denotes lysine lactylation; pan-Kla refers to an assay signal without resolved protein, site, or isomer specificity. H3K18la denotes histone H3 lysine 18 lactylation; K_D_-la denotes D-lactyllysine. IHC, immunohistochemistry; MS, mass spectrometry. Each readout must be interpreted at the scale it measures.

## 6 Potential therapeutic directions and open questions

The two strands of evidence support distinct intervention hypotheses. *P. aeruginosa* consumes lactate through regulated D- and L-lactate systems and can benefit from lactate-associated acidity (12–14, 66). Separately, 2-AA reprograms host pyruvate handling, autophagy, and lipid metabolism (18, 19). Evidence maturity differs across host-cell contexts. The airway study combines bacterial mutation, metabolic rescue, downstream perturbation, cell-function assays, and *in vivo* outcomes (17). Macrophage tolerance has substantial full-length support (18, 19, 80–82), but the proposed lactate/H3K18la extension remains an abstract-level, T3 hypothesis (20). Studies of *S. aureus* biofilms and sepsis provide context without resolving this *P. aeruginosa*-specific gap (114, 122–125).

The epithelial lactate/H3K18la decrease was reported in a full-length study, whereas the proposed macrophage increase rests on a supplement abstract. Different bacterial perturbations, cell types, and experimental systems underlie these reports. The responses have not been shown to coexist within one infected tissue and should not be presented as a validated opposing pair. **Figure 5** summarizes these host-cell-context-dependent responses while distinguishing the established epithelial evidence from the proposed macrophage lactate/H3K18la pathway.

**Figure 5:**
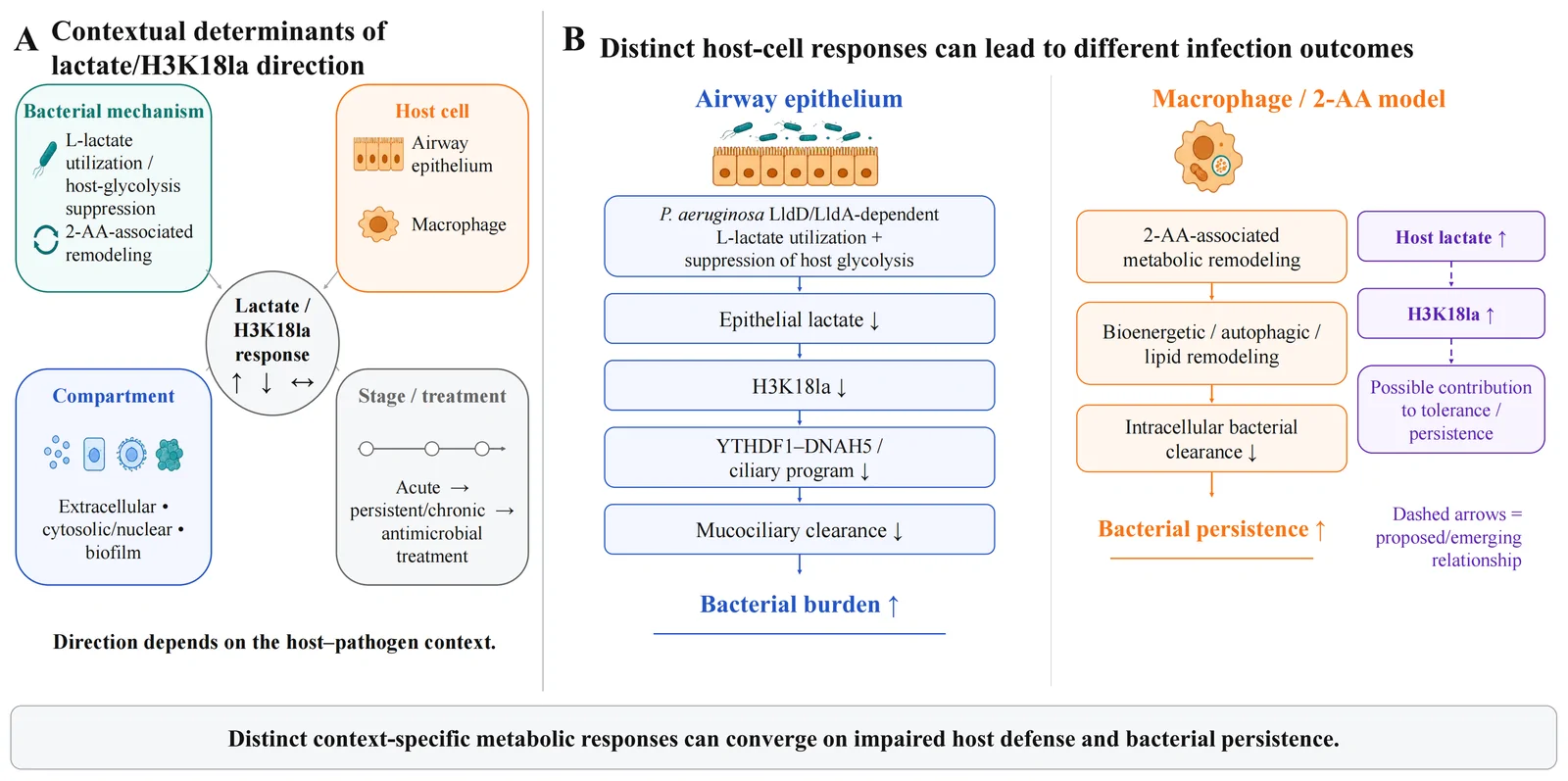
Host–pathogen context shapes lactate/H3K18la responses and their consequences for host defense and bacterial persistence. **(A)** The direction of lactate and H3K18la responses is shaped by bacterial mechanism, host-cell type, anatomical or subcellular compartment, and infection stage or treatment. **(B)** In airway epithelial cells, *Pseudomonas aeruginosa* LldD/LldA-dependent L-lactate utilization and suppression of host glycolysis are associated with lower lactate and H3K18la. These changes accompany impaired YTHDF1–DNAH5-dependent ciliary defense, reduced mucociliary clearance, and increased bacterial burden (17). In macrophages, 2-AA-associated bioenergetic, autophagic, and lipid-metabolic remodeling impairs intracellular clearance and promotes persistence (18, 19). Dashed arrows identify the separate research hypothesis linking increased macrophage lactate and H3K18la to tolerance and persistence (20). This hypothesis derives from a supplement abstract and awaits full-length reporting and independent validation. The epithelial and macrophage lactate/H3K18la responses have not been demonstrated together in one infected tissue. H3K18la, histone H3 lysine 18 lactylation; 2-AA, 2-aminoacetophenone; YTHDF1, YTH N^6^-methyladenosine RNA-binding protein 1; DNAH5, dynein axonemal heavy chain 5.

The following potential therapeutic directions require dedicated preclinical evaluation in the intended infection setting; clinical benefit cannot be inferred from the mechanisms summarized here. **Table 3** distinguishes their supporting evidence and trade-offs. Inhibiting bacterial L-lactate utilization could preserve host substrate and weaken early intracellular survival (13, 14). However, *P. aeruginosa* can switch carbon sources, and the consequences of increasing host lactate remain untested.

**Table 3:** Potential therapeutic directions at the lactate interface, their supporting mechanisms, and their trade-offs.

Intervention	Mechanistic basis	Potential host benefit	Risk or possible benefit to the pathogen	Evidence tier
Inhibit bacterial L-lactate utilization (LldP/LldD/LldA)	Lactate is a preferred bacterial substrate and supports early intracellular survival (13, 14)	May preserve host substrate and weaken early intracellular survival	Metabolic flexibility allows a switch of carbon source; downstream consequences of a higher host lactate pool are untested	T2
Buffer airway acidity/lower the accumulated extracellular pool	Acidic conditions promote OMV release, HHQ-mediated LL-37 resistance, and OMV-driven IRF3–IFN-β activation (66, 67)	May restore cathelicidin activity and limit the interferon response	pH also governs mucus properties, epithelial killing, and the wider microbial community (43, 77)	T2
MCT-directed intervention	Transport separates extracellular exposure from intracellular availability (33, 34, 38)	Could modulate host intracellular lactate	Not cell-selective; broad effects on host cell metabolism	T3
p300/HDAC-directed intervention	Acts at the modification step (17, 105, 106)	Could alter modification-associated host responses; net benefit untested	Not lactylation-specific; affects acetylation and transcription broadly	T3
Reverse 2-AA-associated tolerance (MvfR-directed compounds; ATP or fatty acyl-CoA rescue)	MvfR/2-AA drives tolerance and impaired clearance (18, 19, 60)	May improve intracellular clearance, as observed in experimental models	Tolerance also limits injury; reversal may worsen inflammatory tissue damage (80–82)	T2
Restore epithelial lactate	Lactate rescue restored ciliary defense in the airway model (17)	May support mucociliary function, as observed in the airway model	The same substrate feeds the pathogen (13, 14)	T2
Extracorporeal removal of quorum-sensing molecules (CytoSorb)	Circulating signaling molecules associated with lactate; selected molecules decreased early during adsorption (68)	Potential reduction of circulating bacterial signals; host-defense benefit unproven	Observational pilot (12 patients); mixed infections with a *P. aeruginosa* bloodstream subgroup; later rebound; no established causal lactate effect or efficacy	T3 (contextual clinical evidence)

Evidence tiers describe the mechanistic basis, as defined in **Table 1**, rather than validated therapeutic efficacy. All benefits listed are potential and require evaluation in the intended infection setting.

Buffering airway acidity or lowering accumulated extracellular lactate targets the local chemical environment. Raising pH may restore cathelicidin activity and limit the OMV-driven interferon response (66, 67). Yet pH also influences mucus properties, epithelial killing, and the wider microbial community (43, 77). MCT-directed intervention could alter extracellular exposure and preserve intracellular substrate (33, 34, 38). Its broad metabolic effects and lack of cell selectivity remain important constraints.

p300- or HDAC-directed intervention targets modification-associated processes, but these enzymes also regulate acetylation and transcription beyond H3K18la. The airway C646 experiment therefore supports mechanistic interpretation rather than therapeutic validation (17, 105, 106). MvfR-directed compounds or rescue of bioenergetic and lipid nodes with ATP or fatty acyl-CoA improved clearance in experimental models (18, 19, 60). Reversing tolerance could nevertheless worsen inflammatory injury (80–82). These interventions do not validate the abstract-level macrophage lactate/H3K18la hypothesis.

Lactate supplementation illustrates the central trade-off. It supported ciliary defense in the airway model, but the same substrate feeds *P. aeruginosa* (13, 14, 17). A useful intervention would need to distinguish host-cell rescue from pathogen nutrition. Extracorporeal removal of quorum-sensing molecules is another exploratory direction (68). The small observational CytoSorb study supports molecular removal, while infection-specific benefit and causal effects on lactate remain unresolved.

The choice among potential therapeutic directions depends on cell-specific function, rather than the direction of lactate or H3K18la change alone. Epithelial studies should connect ciliary motion to transport. Macrophage studies should assess intracellular clearance, inflammatory output, and tissue injury together (17–19). Clinical findings reinforce this caution. Sputum lactate fell after intravenous antibiotics in one cohort, but remained stable and associated with neutrophils in another (8, 11). It therefore cannot be treated as a simple treatment-response marker.

Three questions merit priority. First, do epithelial depletion and the hypothesized macrophage lactate/H3K18la increase coexist within one infected tissue? Testing this requires cell-resolved host measurements alongside localization of bacterial *lld* expression or 2-AA activity. Second, how do bacterial utilization, pathogen-induced host production, and treatment redistribute lactate across infection stages and biofilm microenvironments (46, 126)? Third, does the acidification–OMV–LL-37 pathway operate across other *P. aeruginosa*-infected airways, and can buffering reverse it (66)? Spatial analysis and isotope tracing could distinguish host and bacterial carbon while linking its fate to mucociliary transport, macrophage clearance, and bacterial burden.

## 7 Conclusion

*P. aeruginosa* and its host share lactate, but bacterial activity and host-cell context determine its availability and effects. The bacterial strand connects lactate to growth, intracellular survival, biofilm organization, and acidity-associated resistance to antimicrobial peptides. The host strand connects bacterial utilization and epithelial glycolytic suppression to impaired ciliary defense. Separately, 2-AA-associated macrophage reprogramming can promote tolerance and persistence. Histone lactylation extends this framework, with the most complete evidence in the airway model. The proposed macrophage lactate/H3K18la branch remains a research hypothesis requiring full-length reporting and independent validation. Potential interventions must be judged by cell-specific function and the competing demands of host defense and pathogen nutrition. The priority is to resolve lactate sources and fates, defined modification changes, and defense outcomes within the same infection setting.
